# Understanding the Relationship between Food Security and Mental Health for Food-Insecure Mothers in Virginia

**DOI:** 10.3390/nu14071491

**Published:** 2022-04-02

**Authors:** Rachel A. Liebe, Leah M. Adams, Valisa E. Hedrick, Elena L. Serrano, Kathleen J. Porter, Natalie E. Cook, Sarah A. Misyak

**Affiliations:** 1 Department of Human Nutrition, Foods, and Exercise, Virginia Tech, Blacksburg, VA 24061, USA; nelsonr2@vt.edu (R.A.L.); vhedrick@vt.edu (V.E.H.); serrano@vt.edu (E.L.S.); 2 Department of Psychology and Women & Gender Studies Program, George Mason University, Fairfax, VA 22030, USA; ladamse@gmu.edu; 3 Department of Public Health Sciences, School of Medicine, University of Virginia, Christiansburg, VA 24073, USA; kjp9c@virginia.edu; 4 Department of Population Health Sciences, Virginia Tech, Blacksburg, VA 24061, USA; necook@vt.edu

**Keywords:** food security, mental health, maternal health, social support

## Abstract

Food insecurity, which disproportionately impacts mothers, can have chronic consequences on physical and mental health. There is a relationship between food insecurity and mental health, but the relationship’s mechanisms are unclear. This study aimed to understand how mental health outcomes differ by food insecurity severity and race among Virginia mothers. A cross-sectional survey employed previously validated food security status measures, physical and mental health, social support, and food coping strategies. Results were analyzed using descriptive statistics, Spearman’s rank-order correlations, linear regression, and chi-squared with effect sizes. Overall, respondents (*n* = 1029) reported worse mental health than the U.S. average (44.3 ± 10.1 and 50, respectively). There was a large effect of food security on mental health (*d* = 0.6), with worse mental health outcomes for mothers experiencing very low food security (VLFS) than low food security (LFS; *p* < 0.001). There was a small effect of race on mental health (φ_c_ = 0.02), with Black mothers having better mental health than White mothers (*p* < 0.001). Compared to mothers experiencing LFS, mothers experiencing VLFS had less social support (*d* = 0.5) and used more food coping strategies, especially financial strategies (*d* = −1.5; *p* < 0.001). This study suggests that food-insecure mothers experience stressors and lack adequate social support, which is even more distinct for mothers experiencing VLFS.

## 1. Introduction

Food insecurity and mental health are related public health issues that disproportionately impact mothers and were exacerbated by inequities in unemployment [1] during the COVID-19 pandemic. In 2019, over one-quarter (28.7%) of single mother households experienced food insecurity compared to 13.6% of all households with children and 10.5% of all households [2]. There are numerous physical consequences of food insecurity, including a higher risk of obesity, diabetes, hypertension, and other chronic diseases [3,4]. This is particularly concerning as food-insecure mothers often reduce their own intake as a food coping strategy in an effort to avoid limiting the dietary intake of children [5].

Food insecurity is linked to an increased risk of developing mental illness, poor mental health, depression, anxiety, psychological distress, and sleep disturbances [5,6,7,8,9,10]. Coupled with higher rates of food insecurity among mothers, there is also evidence of gender inequities in mental illness and treatment, with mothers experiencing mental illness (22.8%) more often than fathers (12.4%) [11]. Mental health care access and cost can be major barriers to treatment, especially for individuals with low income and without adequate health insurance. In a recent study of California residents, nearly 90% of women who were identified as having mild psychological distress were not receiving adequate treatment [12,13].

### 1.1. Relationship between Food Insecurity and Mental Health

There is evidence to suggest a relationship between mental health and food security for several populations. An association between depression and food insecurity has been found for the general population, low-income caregivers, older adults, and people who are HIV+ [14,15]. Additionally, several studies have suggested that the risk for symptoms of mental illness increases with increasing severity of food insecurity, with the greatest risk for those who are experiencing hunger associated with their food insecurity [5,16,17,18]. For people experiencing food insecurity, the risk of worsening mental health is more pronounced for women than men [7,8,19].

Food insecurity can also impact members of a household differently. For example, children are often shielded from the effects of food insecurity when possible. However, there is limited evidence on the impact of food insecurity on the mental health of mothers [4]. Existing literature does suggest that mothers experiencing food insecurity are at a greater risk for symptoms of mental illness than food-secure mothers [9]. Additionally, for mothers currently experiencing food insecurity, those with depression disorders are more likely to become chronically food insecure than those not experiencing a depression disorder [20]. Understanding the effect of food security status on mental health for mothers has been under-investigated despite the potential downstream impact of food insecurity, and the associated stress on parental behavior and the mental health of children in the household [10].

While there is evidence suggesting a relationship between food insecurity and poor mental health, factors influencing this relationship have not been well investigated [4,5]. Factors that have been proposed as part of the mechanism of this relationship include physical health, social support, and behavioral food coping strategies [4,5,21]. The role of these factors in the relationship between food insecurity and mental health has primarily been explored individually for each factor rather than evaluating the role of multiple factors. There is evidence to suggest that worsening physical health is associated with both food insecurity and mental health [3,4,22]. Additionally, social support has been shown to reduce the risk of depression symptoms among those with food insecurity. However, the effect of social support was reduced as food insecurity became more severe [23,24,25,26,27]. Lastly, having to rely on food coping strategies that involve acquiring food in socially undesirable ways can cause stress, increase symptoms of mental illness, cause social isolation, and worsen quality of life [8,17,28,29,30]. However, the relationship between these factors and mental health has not been well established for mothers experiencing food insecurity.

### 1.2. Purpose of the Study

Despite existing evidence on the presence of a relationship between food insecurity and poor mental health, there are no interventions targeting this relationship [5]. A more robust understanding of the specific mechanisms of the relationship between mental health and food insecurity is needed to inform the development of interventions. Furthermore, mothers are integral to the reduction of household food insecurity as they often make household food decisions [5], yet the relationship between food insecurity and mental health has not been well explored in this population. The aim of this study was to understand the differences in the relationship between mental health and food insecurity among mothers with low and very low food security (i.e., high and very high food insecurity) and to elucidate the potential factors, including physical health, social support, and food coping strategies, influencing this relationship.

## 2. Materials and Methods

We utilized a cross-sectional design where eligible participants completed an online survey. Consent was implied based on the respondent continuing the survey beyond the information sheet. This study was approved by the Virginia Tech IRB.

### 2.1. Respondents

The target population was mothers with low income in Virginia who reported being food insecure. Virginia was chosen as the study location based on the research team’s interest in utilizing the results to inform strategies to address existing disparities in the state. To be eligible for the survey, respondents had to be over the age of 18, live in Virginia, identify as a woman or non-binary, have children under the age of 18 living in the household with them, have an income below the federal poverty level for their household size, speak English, and be experiencing food insecurity. The children were not required to be the biological child of the respondent to accommodate current trends of grandparents serving as primary caregivers or adopting grandchildren [31]. Respondents not meeting the eligibility criteria were excluded from the study.

### 2.2. Survey Development

The survey was developed using existing validated instruments for all measures other than demographics. Individual measures are detailed below. The survey was reviewed by food insecurity and mental health experts for face validity. A pre-test was conducted with fifty participants prior to the main data collection period. The pretest resulted in only minor adjustments to survey flow.

### 2.3. Survey Administration

The survey was administered from 9 August 2021 to 27 October 2021 through Qualtrics. Qualtrics reached potential respondents through existing survey panels, email listservs, and notifications on dedicated applications. The wording of survey invitations intentionally did not include details about the survey to reduce self-selection bias. These panels comprised individuals who consented to participate in surveys for incentives ranging from USD 2 to 4 per survey. Respondents’ identities were verified by third-party sources before joining the survey panel and any respondents completing the survey in less than half of the median response time were removed to improve data integrity. Any response not meeting the eligibility criteria was excluded, and responses were collected until a minimum of 1000 eligible responses were obtained.

### 2.4. Measures

*Sociodemographic Information.* Age, gender identity, race, ethnicity, number of children in the household, household size, income, and participation in nutritional assistance programs were collected.

*Food Security Status.* The United States Department of Agriculture (USDA) Household Food Security Module was used to assess respondents’ food security status over the past twelve months [32]. The validated, 18-item tool was developed to assess food security status for both adults and children in the household. For each item, responses are coded as affirmative or negative. Affirmative responses are assessed as a score of 1, whereas negative responses are scored as 0. For households with children, scores range from 0 to 18. Scores are categorized as high food security (0), marginal food security (1–2), low food security (LFS; 3–7), and very low food security (VLFS; 8–18) [32].

*Physical Health and Diet Quality.* Physical health was assessed using validated, two single-item questions to estimate the respondents’ perceived overall physical health and diet quality [33]. The physical health question asks respondents to rate their overall physical health on a Likert-type scale from 1 to 5 (poor to excellent). The diet quality question asks respondents to rate their diet quality on a Likert-type scale from 1 to 5 (poor to excellent) [34]. There is no specific timeframe indicated for the physical health or diet quality questions. Both items have been shown to have construct validity and can act as proxies for more robust measurement tools [33,34]. Single-item scales were chosen to reduce the response burden on participants.

*Mental Health.* Five measures were used to assess mental health. Overall mental health was assessed with the validated Patient Reported Outcomes Measurement Information System (PROMIS) Global Mental 2a Scale v1.2 [35]. This two-item measure uses a Likert-type scale from 1 to 5 (poor to excellent). There is no specific timeframe indicated for these questions. Scores are then summed, and the raw scores are converted to T-scores using the National Institutes of Health (NIH) Toolbox, which are standardized to the United States (U.S.) population [36]. A T-score less than 50 indicates worse than average overall mental health. T-scores can range from 25.8 to 64.6. Ten units on the T-score scale indicate one standard deviation from the mean [36].

Symptoms of anxiety were assessed for the past seven days with the PROMIS Emotional Distress v1.0—Anxiety—Short Form 4a [37]. This four-item scale is measured on a Likert-type scale from 1 to 5 (never to always). Scores are summed and converted to T-scores following the procedure used for overall mental health. T-scores can range from 40.3 to 81.6 [36].

Symptoms of depression were measured for the past seven days with the PROMIS Emotional Distress v1.0—Depression—Short Form 4a [37,38]. This four-item, validated scale is scored and interpreted like the Anxiety scale above.

Stress over the past month was assessed using the NIH Toolbox Perceived Stress measure for adults [39]. Each item in this ten-item tool is measured on a scale from 1 to 5 (never to very often). Scores are summed and converted to T-scores using the same procedure described for measures above. T-scores can range from 22.7 to 87.1 and are grouped into three categories: low (<40), average (41–59), and high stress (60+).

Life satisfaction was measured using the Satisfaction with Life Scale [40]. This five-item tool uses a Likert-type scale from 1 to 7 (strongly disagree to strongly agree). There is no specific timeframe indicated for this scale. Scores are summed for the five questions and categorized according to the standardized levels. Scores range from 5 to 35 and are categorized into six groups: highly satisfied (30–35), high (25–29), average (20–24), slightly below average (15–19), dissatisfied (10–14), and extremely dissatisfied (5–9) [40].

*Social Support.* A previously validated, modified Duke Social Support Inventory was used to assess social support [41]. This ten-item scale is divided into two subscales, interaction and satisfaction. The interaction subscale contains four questions, and scores range from 4 to 12 and assessed the social interaction the respondent experienced over the past week. Higher scores indicate higher levels of social interaction. The satisfaction subscale contains six questions, with scores ranging from 6 (low to no social support) to 18 (high social support). The satisfaction subscale assessed how satisfied the respondent was with their social support and was not assessed over a specific timeframe. Total scores for the scale range from 10 to 30, with 30 being the highest level of social support [41].

*Food Coping Strategies.* The Hunger Coping Scales were used to assess strategies for coping with food insecurity over the past month [21]. The 17-item scale includes three subscales: tradeoffs, financial, and rationing. For tradeoffs, the question asks respondents how often they choose between paying for food and other categories of expenses (i.e., rent/mortgage) on a scale from 1 to 5 (never to always). The average of the five questions is used to determine the total tradeoff score, with a higher score indicating more frequent usage of tradeoffs [21]. The questions for the financial and rationing subscales are binary, with 1 indicating the participant had used the strategy in the past month and 0 indicating they had not. For each of these two subscales, scores are summed and can range from 0 to 5. A score of 5 indicates a greater usage of coping strategies in that subscale [21]. There were two additional coping strategies that were assessed but were not part of the subscales: (1) attending an event only to eat and (2) removing spoiled parts of produce. These independent questions were also binary, with 1 indicating the participant had used the strategy in the past month and 0 indicating they had not [21].

### 2.5. Data Analysis

Descriptive statistics were conducted to summarize demographic data for the survey sample and to obtain means ± standard deviation for each survey measure. Spearman’s rank-order correlation coefficients were conducted for social support, food coping strategies, global mental health, anxiety, and depression. These same variables were compared by race and food security status using simple linear regression. Effect sizes were calculated using partial eta squared and Cohen’s d for race and food security status, respectively. For partial eta squared, greater than 0.01 was considered small, 0.06–0.13 was medium, and greater than 0.14 was large. For Cohen’s d, effect sizes were interpreted as follows: small (0.20–0.49), medium (0.50–0.79), and large (greater than 0.80) [42]. Post-hoc analyses using pairwise *t*-tests were conducted for statistically significant relationships by race. Chi-squared tests were used to calculate differences between observed and expected frequencies in a contingency table for stress, life satisfaction, physical health, diet, race, and food security status. Effect sizes for chi-squared were calculated using Cramer’s V. Effects were interpreted as negligible (less than 0.20), small (0.20–0.39), medium (0.40–0.49), and strong (greater than 0.50) [43]. Post-hoc analyses were conducted by evaluating cell residuals for significant relationships. Bonferroni corrections were applied to each test to compensate for the increase in type I error associated with multiple tests.

## 3. Results

### 3.1. Respondents’ Demographic Characteristics

There was a total of 1029 responses. All respondents were food insecure; 232 respondents were experiencing LFS and 797 were experiencing VLFS. Respondents ranged in age from 18 to 80 years old and were living in a household with one to nine children. Approximately two-thirds of respondents reported living with a spouse or unmarried partner. Household incomes ranged from USD 0 to 80,000. Respondents reported participating in 2.0 ± 1.3 assistance programs in the past year, with over half of respondents reporting that they received SNAP benefits. Overall, 65.0% (*n* = 673) of respondents identified as White, 23.4% (242) as Black or African American, and 9.7% (100) as Hispanic/Latino. More than three-quarters of respondents reported their educational attainment as some college or less. (See Table 1).

Most respondents identified their overall health as good to excellent (66.5%), yet just under half of respondents described their diet quality as poor to fair (48.8%). The T-scores for global mental health ranged from 25.8 to 64.6, where scores below 50 indicate worse mental health than the U.S. average. (See Table 1) The T-scores for symptoms of anxiety ranged from 40.3 to 81.6 and depression ranged from 41 to 79.4, where scores above 50 indicate greater presence of symptoms than the U.S. average. More than half of respondents reported a high level of stress (57.5%, 592). More than half of respondents also reported below-average life satisfaction (58.1%, 598).

The interaction social support sub-scores ranged from 4 to 12 (out of a possible range of 4 to 12), while the satisfaction sub-scores ranged from 6 to 18 (6–18), and the total social support scores ranged from 10 to 30 (10–30). Respondent usage of food coping strategies is outlined in Table 1. Respondents’ usage of both financial and rationing coping strategies ranged from 0 to 5, out of a possible five coping strategies in each category.

### 3.2. Associations between Mental Health, Social Support, and Food Coping Strategies

Table 2 shows correlations between mental health, social support, and food coping outcomes. Within social support, total social support was strongly correlated with both satisfaction (r_s_ = 0.90) and interaction social support (r_s_ = 0.77, (*p* < 0.001)). All types of social support (total, interaction, and satisfaction) were positively correlated with global mental health (r_s_ ranged from 0.27 to 0.44) and negatively correlated with greater anxiety (r_s_ ranged from −0.16 to −0.38) and depression symptoms (−0.24 to −0.44; (*p* < 0.001)). Food coping strategies (tradeoffs, financial, and rationing) were negatively correlated with total social support and satisfaction, but not interaction social support (*p* < 0.001). The food coping strategies were negatively correlated with global mental health for tradeoffs, financial, and rationing (*p* < 0.001). Increased use of food coping strategies was positively correlated with increased anxiety (r_s_ ranged from 0.40 to 0.41) and depression symptoms (0.39 to 0.43; *p* < 0.001).

### 3.3. Impact of Food Security Status

Respondents experiencing VLFS reported significantly less total social support (*d* = 0.5), interaction support (0.3), and satisfaction with social support (0.6) than respondents experiencing LFS (*p* < 0.001). Additionally, respondents with VLFS used more food coping strategies than those with LFS (ranging from −0.9 to −1.5 for the types of food coping strategies, *p* < 0.001). There were no significant differences in the number of people in the household or income between the VLFS and LFS groups. Global mental health was worse for respondents experiencing VLFS than LFS (0.6, *p* < 0.001). Additionally, symptoms of anxiety and depression were more prevalent in those experiencing VLFS (−0.8) compared to LFS (−0.8; *p* < 0.001). (See Table 3).

Table 4 shows the differences in stress and life satisfaction by food security status. The effect size of food security on stress was small, φ_c_ = 0.3. There were more respondents with VLFS who reported high stress (64.4%, 513) than expected (*p* < 0.001). Additionally, there were fewer respondents with LFS who reported high stress (34.1%, 79) than expected (*p* < 0.001). There was also a small effect of food security on life satisfaction (φ_c_ = 0.3). Respondents with VLFS reported being extremely dissatisfied with their life more often (23.7%, 152) than expected (129.3, *p* < 0.001). Respondents with LFS reported being extremely dissatisfied with their life less often (*n* = 15, 6.5%) than expected based on the chi-squared test (37.7, *p* < 0.001).

### 3.4. Impact of Race

There were no significant differences in food security status, social support, and food coping strategies by race (see Table 3). Respondents who identified as Black reported significantly greater global mental health (46.5 ± 10.5) compared to those who identified as White (43.4 ± 9.9, *p* < 0.001). There was no significant difference for global mental health between people who identified as other races and either group. The effect of race on mental health was small (η^2^ = 0.02). Similarly, people who identified as Black reported fewer symptoms of anxiety and depression compared to people who identified as White. Again, the effect size was small (0.01). Overall, 423 (63.1%) White respondents reported high stress, which was significantly more than expected (*p* < 0.001). Additionally, there were fewer White respondents (33.6%) who reported average stress (*n* = 225 observed, *n* = 260.4 expected, *p* < 0.001). There were significantly fewer Black respondents (43.9%, 105) who reported higher stress than expected (*p* < 0.001). There were no significant differences from what we expected for the Other Race group. The effect of race on stress was small (φ_c_ = 0.1). There was also no significant difference in life satisfaction by race (Table 4).

## 4. Discussion

The findings from this study suggest a relationship between VLFS and negative mental health outcomes for mothers. There were no significant differences in income or household size between the LFS and VLFS groups. This suggests that there are other factors that are likely contributing to the severity of food insecurity. Based on the findings of this study, factors that may be involved in the mechanism of this relationship include social support and food coping strategies.

Mothers in this study reported experiencing anxiety and depression symptoms at more than one standard deviation higher than the mean for U.S. adults, which is consistent with a 2020 study indicating a greater prevalence of anxiety and depression symptoms among mothers during the COVID-19 pandemic [44]. Mothers also reported high levels of stress, a significant risk factor for maternal depression [44]. However, this is not unique to the COVID-19 pandemic. In 2003, there was evidence of an association between worsening food insecurity and anxiety and depression disorders [45]. This trend of worsening mental health among mothers experiencing more severe food insecurity is concerning because low-income mothers often face significant barriers to seeking treatment: transportation, cost, childcare, stigma, lack of trust in healthcare providers, and lack of recognition of the need for treatment [44,46]. Addressing these barriers is needed for mothers to seek treatment, as not receiving treatment can have negative consequences for mothers and children [44].

Overall, food coping strategies were associated with worse mental health outcomes and higher levels of stress. Respondents in the present study utilized slightly fewer tradeoff strategies and more financial and rationing strategies than the respondents in a study of low-income adults by Pinard et al. (2014) [21]. While the demographics of the sample in this study differed from the sample in the study by Pinard et al., they found an increase in tradeoffs associated with worsening food insecurity, which is consistent with the findings in this study. Previous research has identified these strategies as manifestations of the experience of food insecurity, which is consistent with the high usage of these strategies in this study and the correlation with worsening mental health outcomes [21]. Having to rely on these strategies to acquire food may be a stressor for respondents, although the directionality of the relationship was not assessed here. Future research should determine effective strategies for mitigating hunger and explore potential coping strategies for dealing with the stress associated with food insecurity.

Respondents who identified as Black had better overall mental health and fewer symptoms of anxiety and depression than those who identified as White, though the effect size of race was small. This is not consistent with existing literature that suggests similar levels of anxiety and depression across racial groups [47]. Additionally, fewer people who identified as Black reported high stress than expected in this study. This is, again, not consistent with existing literature suggesting that people who identify as Black often experience higher levels of chronic stress, including stress from systemic racism [48]. Future research should explore factors that may improve resiliency to worsening mental health outcomes when a person is experiencing food insecurity.

The findings of this study suggest that people with VLFS experienced an environment that was generally stressful and lacked sufficient social support, which is associated with worse mental health. This is supported by the medium and large effects of food security on mental health outcomes in this study. More than half of all respondents were experiencing a high level of perceived stress, which is severe enough to potentially necessitate clinical intervention [39]. Notably, a study of parents and other caregivers early in the COVID-19 pandemic (April 2020) found that respondents were experiencing moderate stress [49]. While the population for that study differed from the present study, this suggests that food insecurity serves as a stressor. While people with LFS were also experiencing significant stress, perceived stress was higher for mothers experiencing VLFS. Respondents experiencing VLFS also relied on more food coping strategies compared to those experiencing LFS. This finding was consistent with previous literature from before the COVID-19 pandemic demonstrating that individuals with lower food security status, especially those facing hunger, were more likely to employ food coping strategies than those with higher food security status [21]. Efforts to mitigate some of these disparities among people with VLFS are needed. However, it is important to remember that people with LFS are still experiencing significant symptoms of anxiety, depression, and stress, and these should also be addressed.

There were several limitations of this study. First, invited participants were randomly selected from all eligible panel members within Virginia; therefore, the sample may not be generalizable to the entire state or beyond. Furthermore, the neighborhood a person lives in and the degree to which it is rural or urban can influence several of the factors surveyed here, such as food security and social support [50]. Urbanicity was not assessed in this study. Despite these limitations, the results of this study provide insight into the relationship between mental health and food security status. Secondly, the original quota for non-White respondents was 50% of the sample to allow for robust comparisons between racial and ethnic groups. However, we were unable to reach this quota during the time horizon of the study. Despite this, the sample of respondents obtained had a similar racial and ethnic demographic profile as the state of Virginia [51]. Finally, data collection took place during the ongoing COVID-19 pandemic. To determine the effect of the pandemic, outcomes ideally would have been compared to previous literature prior to the pandemic. Due to the extremely limited literature on this topic with food-insecure mothers, this was not feasible for some measures, such as social support. However, the results of this study still provide a deeper understanding of some of the factors influencing food security status and mental health outcomes.

Mothers represent a strong potential target for intervention related to improving both food security and mental health, as they often make decisions related to the home food environment [5]. Improving maternal mental health and food security can directly address child food security, which is critical for meeting the Healthy People 2030 goal of eliminating VLFS in children [52]. To this end, future research should identify intervention strategies that can be used by mothers who are food insecure to cope with stress and improve overall mental health. Secondly, a framework should be developed to explain the relationship between food security and mental health and factors that may be impacting this relationship. This can be used to develop future interventions that address these factors to improve food security and mental health for mothers with low income, as well as guide future research. As the research in this area continues to develop, healthcare professionals can incorporate more considerations around the relationship between food insecurity and mental health into their practice to better meet the needs of food-insecure mothers.

## Figures and Tables

**Table 1:** Description of survey respondents’ demographics, physical health, mental health, social support, and coping strategies (*n* = 1029).

Variables	Mean ± SD or *n* (%)
**Respondent Demographics**	
Age, Years	34.0 ± 9.8
Education	
Less than High School	70 (6.8)
High School or GED	410 (39.8)
Some College	319 (31.0)
Associate’s	119 (11.6)
Bachelor’s	85 (8.3)
Postgraduate	26 (2.5)
Race	
White	673 (65.4)
Black or African American	242 (23.5)
Asian	26 (2.5)
Two or More Races	52 (5.1)
Other	43 (4.2)
Ethnicity	
Hispanic	100 (9.7)
Number of People in Household	4.2 ± 1.7
Number of Children	2.0 ± 1.1
Child Age, Years	7.6 ± 5.2
Household Members	
Spouse	386 (37.5)
Unmarried Partner	263 (25.6)
Child Under 6	542 (52.7)
Child Over 6	603 (58.6)
Parent	174 (16.9)
Sibling	96 (9.3)
Relative	78 (7.6)
Housemate	35 (3.4)
Renter	6 (0.6)
Other	28 (2.7)
Childcare Assistance	
Spouse	353 (34.3)
Unmarried Partner	239 (23.2)
Older Child	125 (12.1)
Parent	265 (25.8)
Sibling	77 (7.5)
Relative	98 (9.5)
Housemate	18 (1.7)
Other	111 (10.8)
Annual Household Income, USD	25,000 ± 16,032
Assistance Program Usage ^1^	
SNAP	606 (58.9)
WIC	242 (23.5)
NSLP/SBP	389 (37.8)
Head Start	40 (3.9)
TANF	80 (7.8)
Food Banks	280 (27.2)
TEFAP	35 (3.4)
Public Housing	74 (7.2)
USDA Summer Meals	59 (5.7)
SNAP-Ed	101 (9.8)
None	179 (17.4)
Change in Assistance Program Benefits	
Started	46 (4.5)
Increase	349 (33.9)
No Change	460 (44.7)
Decreased	93 (9.0)
Lost	81 (7.9)
Food Security Status ^2^	
Low	232 (22.5)
Very Low	797 (77.5)
**Physical Health ^2^**	
Overall Physical Health	
Poor	69 (6.7)
Fair	275 (26.7)
Good	384 (37.3)
Very Good	202 (19.6)
Excellent	99 (9.6)
Health Conditions	
Obesity	221 (21.5)
High Blood Pressure	274 (26.6)
Heart Disease	31 (3.0)
Diabetes	73 (7.1)
Cancer	25 (2.4)
None	567 (55.1)
Diet Quality	
Poor	145 (14.1)
Fair	357 (34.7)
Good	349 (33.9)
Very Good	123 (12.0)
Excellent	55 (5.3)
**Mental Health ^2^**	
Overall Mental Health ^3^	44.3 ± 10.1
Anxiety Symptoms ^3^	61.2 ± 9.5
Depression Symptoms ^3^	60.3 ± 10.4
Stress	
Low	37 (3.6)
Average	400 (38.9)
High	592 (57.5)
Life Satisfaction	
Extremely Dissatisfied	167 (16.2)
Dissatisfied	198 (19.2)
Slightly Below Average	233 (22.6)
Average	226 (22.0)
High	118 (11.5)
Very High	87 (8.5)
**Social Support ^2^**	
Total Social Support ^4^	19.9 ± 4.6
Interaction Sub-Score ^4^	7.6 ± 2.0
Satisfaction Sub-Score ^4^	12.3 ± 3.4
**Food Coping Strategies ^2^**	
*Tradeoffs Total* ^5^	2.0 ± 1.0
Medicine	1.9 ± 1.1
Utilities	2.3 ± 1.3
Rent/Mortgage	2.2 ± 1.3
Transportation	2.2 ± 1.3
Education	1.6 ± 1.1
*Financial*	
Asked friends and family for food or money for food	537 (52.1)
Sold food or pawned any personal property	302 (29.5)
Skipped paying bills to buy food	515 (50.0)
Bought the cheapest food available	861 (83.4)
Avoided buying expensive foods, e.g., fruits/vegetables	689 (66.8)
Mean = 2.80 (SD = 1.66), 5 items	
*Rationing*	
Locked up or hid food to save it	327 (31.8)
Stretched food by limiting	557 (54.1)
Avoided having guests to avoid serving food	543 (52.8)
Eaten as much as possible when food is available	483 (46.9)
Eaten meals or snacks after children finished	620 (60.3)
Mean = 2.44 (SD = 1.72), 5 items	
*Independent Questions*	
Visited a social or community event just to eat	364 (35.4)
Removed spoiled parts from fruits/vegetables	436 (42.4)

^1^ SNAP: Supplemental Nutrition Assistance Program; WIC: Special Supplemental Nutrition Program for Women, Infants, and Children; NSLP/SBP: National School Lunch Program/School Breakfast Program; TANF: Temporary Assistance for Needy Families; TEFAP: The Emergency Food Assistance Program; SNAP-Ed: SNAP Education. ^2^ Tools used to assess measures: Food Security—United States Department of Agriculture Household Food Security Module; Overall Physical Health—one-item Likert-type scale (DeSalvo et al.); Diet Quality—one-item Likert-type scale (Loftfield et al.); Overall Mental Health—Patient Reported Outcomes Measurement Information System (PROMIS) Global Mental 2a Scale v1.2; Anxiety—PROMIS Emotional Distress v1.0—Anxiety—Short Form 4a; Depression—PROMIS Emotional Distress v1.0—Depression—Short Form 4a; Stress—National Institutes of Health Toolbox Perceived Stress for Adults; Life Satisfaction—Satisfaction with Life Scale; Social Support—Modified Duke Social Support Inventory; Food Coping Strategies—Hunger Coping Scales. ^3^ Scores from these scales are converted to T-scores that are standardized to the U.S. population with a mean score of 50. Scores greater than 50 indicate a greater presence of that phenomenon (i.e., greater mental health or anxiety). ^4^ The total social support is out of a possible 30, the interaction subscale is out of 12, and the satisfaction subscale is out of 18. ^5^ Tradeoffs were measured on a scale from 1 to 5, with 1 being never and 5 being always.

**Table 2:** Spearman’s correlations between mental health, social support, and coping outcomes in food-insecure mothers in Virginia (*n* = 1029).

		1 ^2,3^	2	3	4	5	6	7	8	9
1	Total Social Support (SS) ^1^	-	-	-	-	-	-	-	-	-
2	Interaction SS ^1^	0.77 *	-	-	-	-	-	-	-	-
3	Satisfaction SS ^1^	0.90 *	0.44 *	-	-	-	-	-	-	-
4	Tradeoffs—Coping ^1^	−0.17 *	−0.07	−0.20 *	-	-	-	-	-	-
5	Financial—Coping ^1^	−0.24 *	−0.10	−0.28 *	0.61 *	-	-	-	-	-
6	Rationing—Coping ^1^	−0.22 *	−0.10	−0.26 *	0.55 *	0.66 *	-	-	-	-
7	Global Mental Health ^1^	0.43 *	0.27 *	0.44 *	−0.19 *	−0.30 *	−0.26 *	-	-	-
8	Anxiety ^1^	−0.35 *	−0.16 *	−0.38 *	0.41 *	0.41 *	0.40 *	−0.62 *	-	-
9	Depression ^1^	−0.43 *	−0.24 *	−0.44 *	0.39 *	0.43 *	0.40 *	−0.61 *	0.79 *	-

^1^ Tools used to assess measures: Social Support—Modified Duke Social Support Inventory; Food Coping Strategies—Hunger Coping Scales; Overall Mental Health—Patient Reported Outcomes Measurement Information System (PROMIS) Global Mental 2a Scale v1.2; Anxiety—PROMIS Emotional Distress v1.0—Anxiety—Short Form 4a; Depression—PROMIS Emotional Distress v1.0—Depression—Short Form 4a. ^2,^* denotes correlations that are significant at the Bonferroni adjusted *p*-value threshold of 0.0005. ^3^ denotes that correlation has been presented elsewhere in the table or the correlation between a variable and itself.

**Table 3:** Linear regression of social support, food coping strategies, and mental health by race and food security status for food-insecure mothers in Virginia (*n* = 1029).

	Race				Food Security ^5^			
	White (*n* = 673)	Black (*n* = 242)	Other (*n* = 121)	Effect Size ^1^	Low (*n* = 232)	Very Low (*n* = 797)	Sig. ^4^	Effect Size ^2^
Total Social Support (SS) ^5^	19.8 ± 4.6	20.5 ± 4.5	19.8 ± 4.7	<0.01	21.8 ± 4.5	19.4 ± 4.5	**	0.5 (medium)
Interaction SS ^5^	7.5 ± 2.0	7.9 ± 2.0	7.4 ± 1.9	<0.01	8.06 ± 2.0	7.5 ± 2.0	**	0.3 (small)
Satisfaction SS ^5^	12.3 ± 3.4	12.5 ± 3.4	12.3 ± 3.4	<0.01	13.8 ± 3.3	11.9 ± 3.3	**	0.6 (medium)
Tradeoffs—Coping ^5^	2.0 ± 1.0	2.0 ± 1.0	2.1 ± 1.0	<0.01	1.39 ± 0.8	2.2 ± 1.0	**	−0.9 (large)
Financial—Coping ^5^	2.9 ± 1.6	2.6 ± 1.7	2.7 ± 1.7	<0.01	1.9 ± 1.2	3.3 ± 1.4	**	−1.5 (large)
Rationing—Coping ^5^	2.4 ± 1.7	2.4 ± 1.7	2.6 ± 1.7	<0.01	0.9 ± 1.3	2.9 ± 1.6	**	−1.3 (large)
Income ^5^	25,530 ± 15,799	20,769 ± 15,994	27,081 ± 16,000	0.02 (small)	25,081 ± 17,757	24,466 ± 15,460		0.04
People in Household ^5^	4.2 ± 1.8	4.0 ± 1.6	4.4 ± 1.6	0.005	4.3 ± 1.7	4.1 ± 1.8		0.2
Global Mental Health ^3,5^	43.4 ± 9.9 ^a^	46.5 ± 10.5 ^b^	45.1 ± 9.5 ^ab^	0.02 (small)	48.9 ± 10.1	43.0 ± 9.7	**	0.6 (medium)
Anxiety ^3,5^	61.9 ± 9.3 ^a^	59.3 ± 10.4 ^b^	61.3 ± 8.1 ^ab^	0.01 (small)	55.5 ± 10.0	62.9 ± 8.6	**	−0.8 (large)
Depression ^3,5^	61.1 ± 10.3 ^a^	58.3 ± 11.0 ^b^	59.4 ± 8.8 ^ab^	0.01 (small)	54.0 ± 10.5	62.1 ± 9.6	**	−0.8 (large)

^1^ Effect size assessed with a partial eta squared. Effect size greater than 0.01 was considered small. ^2^ Effect size assessed with Cohen’s d. A small effect was defined as greater than 0.3, medium greater than 0.5, and large greater than 0.8. ^3^ The values with different superscript letters (a and b) in a row are significantly different at the adjusted *p*-value when analyzed with pairwise *t*-test. Values with ab are not significantly different from a or b. ^4,^** Correlations with *p*-values < 0.001. ^5^ Tools used to assess measures: Food Security—United States Department of Agriculture Household Food Security Module; Social Support—Modified Duke Social Support Inventory; Food Coping Strategies—Hunger Coping Scales; Overall Mental Health—Patient Reported Outcomes Measurement Information System (PROMIS) Global Mental 2a Scale v1.2; Anxiety—PROMIS Emotional Distress v1.0—Anxiety—Short Form 4a; Depression—PROMIS Emotional Distress v1.0—Depression—Short Form 4a.

**Table 4:** Chi-squared of mental and physical health measures by race and food security status for food-insecure mothers in Virginia (*n* = 1029).

	Race				Food Security (FS) ^3^		
	White (*n* = 673)	Black (*n* = 242)	Other Races (*n* = 121)		Low FS (*n* = 232)	Very Low FS (*n* = 797)	
	*n* ^1^ (%)	*n* (%)	*n* (%)	Effect Size ^2^	*n* ^1^ (%)	*n* (%)	Effect Size ^2^
**Stress ^3^**				0.1			0.3 (small)
High	423 * (63.1)	105 * (43.9)	64 (53.3)		79 * (34.1)	513 * (64.4)	
Average	225 * (33.6)	121 * (50.6)	54 (45.0)		129 * (55.6)	271 * (34.0)	
Low	22 (3.3)	13 (5.4)	2 (1.7)		24 * (10.3)	13 * (1.6)	
**Life Satisfaction ^3^**				0.06			0.3 (small)
Extremely Dissatisfied	120 (17.9)	33 (13.8)	14 (11.7)		15 * (6.5)	152 * (19.1)	
Dissatisfied	136 (20.3)	39 (16.3)	23 (19.2)		26 * (11.2)	172 * (21.6%)	
Slightly Below Average	147 (21.9)	56 (23.4)	30 (25.0)		44 (19.0)	189 (23.7)	
Average	140 (20.9)	57 (23.8)	29 (24.2)		54 (23.3)	172 (21.6)	
High	71 (10.6)	33 (13.8)	14 (11.7)		53 * (22.8)	65 * (8.2)	
Very High	56 (8.4)	21 (8.8)	10 (8.3)		40 * (17.2)	47 * (5.9)	
**Physical Health ^3^**				0.1			0.2 (small)
Excellent	48 * (7.2)	39 * (16.3)	12 (10.0)		40 * (17.2)	59 * (7.4)	
Very Good	133 (19.9)	41 (17.2)	28 (23.3)		59 (25.4)	143 (17.9)	
Good	257 (38.4)	83 (34.7)	44 (36.7)		92 (39.7)	292 (36.6)	
Fair	177 (26.4)	66 (27.6)	32 (26.7)		36 * (15.5)	239 * (30.0)	
Poor	55 (8.2)	10 (4.2)	4 (3.3)		5 * (2.2)	64 * (8.0)	
**Diet Quality ^3^**				0.1			0.2 (small)
Excellent	24 * (3.6)	22 * (9.2)	9 (7.5)		27 * (11.6)	28 * (3.5)	
Very Good	66 (9.9)	33 (13.8)	24 * (20.0)		42 * (18.1)	81 * (10.2)	
Good	245 (36.6)	70 (29.3)	34 (28.3)		89 (38.4)	260 (32.6)	
Fair	236 (35.2)	79 (33.1)	42 (35.0)		58 * (25.0)	299 * (37.5)	
Poor	99 (14.8)	35 (14.6)	11 (9.2)		16 * (6.9)	129 * (16.2)	

^1,^* Outcome significantly differed from the expected value based on a chi-squared test and a significance at the Bonferroni adjusted *p*-value threshold of 0.0025, as analyzed by adjusted standardized residuals. ^2^ Effect size assessed by Cramer’s V. A small effect was defined as greater than 0.2 but less than 0.4. ^3^ Tools used to assess measures: Food Security—United States Department of Agriculture Household Food Security Module; Stress—National Institutes of Health Toolbox Perceived Stress for Adults; Life Satisfaction—Satisfaction with Life Scale; Overall Physical Health—one-item Likert-type scale (DeSalvo et al.); Diet Quality—one-item Likert-type scale (Loftfield et al.).

## Data Availability

The data presented in this study are available on request from the corresponding author. The data are not publicly available due to IRB requirements.
